# The Autophagoproteasome a Novel Cell Clearing Organelle in Baseline and Stimulated Conditions

**DOI:** 10.3389/fnana.2016.00078

**Published:** 2016-07-21

**Authors:** Paola Lenzi, Gloria Lazzeri, Francesca Biagioni, Carla L. Busceti, Stefano Gambardella, Alessandra Salvetti, Francesco Fornai

**Affiliations:** ^1^Department of Translational Research and New Technologies in Medicine and Surgery, University of PisaPisa, Italy; ^2^Istituti di Ricovero e Cura a Carattere Scientifico (I.R.C.C.S.), NeuromedPozzilli, Italy; ^3^Department of Clinical and Experimental Medicine, University of PisaPisa, Italy

**Keywords:** autophagy, proteasome, autophagosome, protein-clearing-pathways, ubiquitination

## Abstract

Protein clearing pathways named autophagy (ATG) and ubiquitin proteasome (UP) control homeostasis within eukaryotic cells, while their dysfunction produces neurodegeneration. These pathways are viewed as distinct biochemical cascades occurring within specific cytosolic compartments owing pathway-specific enzymatic activity. Recent data strongly challenged the concept of two morphologically distinct and functionally segregated compartments. In fact, preliminary evidence suggests the convergence of these pathways to form a novel organelle named autophagoproteasome. This is characterized in the present study by using a cell line where, mTOR activity is upregulated and autophagy is suppressed. This was reversed dose-dependently by administering the mTOR inhibitor rapamycin. Thus, we could study autophagoproteasomes when autophagy was either suppressed or stimulated. The occurrence of autophagoproteasome was shown also in non-human cell lines. Ultrastructural morphometry, based on the stochiometric binding of immunogold particles allowed the quantitative evaluation of ATG and UP component within autophagoproteasomes. The number of autophagoproteasomes increases following mTOR inhibition. Similarly, mTOR inhibition produces overexpression of both LC3 and P20S particles. This is confirmed by the fact that the ratio of free vs. autophagosome-bound LC3 is similar to that measured for P20S, both in baseline conditions and following mTOR inhibition. Remarkably, within autophagoproteasomes there is a slight prevalence of ATG compared with UP components for low rapamycin doses, whereas for higher rapamycin doses UP increases more than ATG. While LC3 is widely present within cytosol, UP is strongly polarized within autophagoproteasomes. These fine details were evident at electron microscopy but could not be deciphered by using confocal microscopy. Despite its morphological novelty autophagoproteasomes appear in the natural site where clearing pathways (once believed to be anatomically segregated) co-exist and they are likely to interact at molecular level. In fact, LC3 and P20S co-immunoprecipitate, suggesting a specific binding and functional interplay, which may be altered by inhibiting mTOR. In summary, ATG and UP often represent two facets of a single organelle, in which unexpected amount of enzymatic activity should be available. Thus, autophagoproteasome may represent a sophisticated ultimate clearing apparatus.

## Introduction

Recently, we described that clearing systems such as the Ubiquitine Proteasome (UP) and Autophagy (ATG) may occur within single morphological entities we named autophagoproteasome (Isidoro et al., 2009; Pasquali et al., 2009; Ferrucci et al., 2011; Natale et al., 2011; Klionsky et al., 2016). Our research team described this organelle in the last decade starting from serendipitous findings, which suggested the expression of both ATG and UP antigens within similar morphological compartments (Castino et al., 2008). At present, when contributing to the “Guidelines for the use and interpretation of assays for monitoring autophagy” (3rd edition) by Klionsky et al. (2016) we shared the definition of autophagoproteasome as “A cytosolic membrane-bound compartment denoted by a limiting single, double or multiple membrane, which contains both LC3 and UP antigens” (Klionsky et al., 2016). When writing the autophagy glossary in the guidelines we contributed to the hypothesis that “The autophagoproteasome may be derived from the inclusion of ubiquitin-proteasome structures within either early or late autophagosomes containing cytoplasmic material at various stages of degradation” (Klionsky et al., 2016). This was based on recent unpublished data. In fact, despite we coined the term autophagoproteasome in the last decade (Pasquali et al., 2009), no specific study was designed to characterize in depth such an organelle. This is crucial since biomedical research is producing increasing evidence on the seminal role played by all clearing pathways in modulating cell survival and disease mechanisms. In fact, ATG and/or UP are involved in a wide range of disorders encompassing tumors (Liang et al., 1999; Qu et al., 2003; Mani and Gelmann, 2005; Bazzaro et al., 2006; Takamura et al., 2011; Huang and Dixit, 2016; Liu and Debnath, 2016), cardiovascular diseases (Nakai et al., 2007; Willis and Patterson, 2013; Wang and Robbins, 2014; Willis et al., 2014; Delbridge et al., 2015), and neurodegenerative disorders (Nedelsky et al., 2008; Bedford et al., 2009; Chu et al., 2009; Madeo et al., 2009; Dantuma and Bott, 2014; Menzies et al., 2015).

The classic definition which is used so far considers these cell clearing pathways to possess distinct biochemical activities which take place within different ultrastructural compartments of eukaryotic cells. In fact, the classic ATG pathway occurs within a double-layered cytosolic vacuole named autophagosome which merges with lysosomes to produce an autophagolysosome which is gifted with a rich enzymatic apparatus to clear various cell cargoes (Klionsky et al., 2016, for a comprehensive definition). In contrast, the classic UP pathway does not imply a well-defined cell organelle. The UP activity is rather considered to occur within dispersed cytosolic domains where UP components represented by two 19S (PA700) subunits and one P20S subunit interact to recognize altered ubiquitinated substrates to provide specific proteolytic clearance (Ciechanover et al., 1981; Hershko et al., 1981; Fornai et al., 2003; Ciechanover, 2006, 2015). In keeping with this, a considerable amount of research studies compared the specificity of each compartment in providing the clearance of specific substrates. Thus, ATG and UP may be further characterized by distinct substrate specificity. In summary, the classic view considers these clearing pathways as distinct depending on various items: compartmentalization, substrate-specificity, enzymatic activities, roles in cell homeostasis.

This manuscript is aimed at characterizing a morpho-functional entity where UP and ATG takes place, which is named autophagoproteasome. The studies are carried out both in baseline conditions and following dose-dependent mTOR inhibition induced by rapamycin. The specimens used to carry out this analysis derive from a variety of experiments from the past 15 years which were implemented by very recent data.

Old specimens were further analyzed to detail and improve early light and electron microscopy data which suggested the occurrence of autophagoproteasome (Fornai et al., 2004; Lazzeri et al., 2007; Ferrucci et al., 2008, 2011; Pasquali et al., 2008; Lenzi et al., 2012).

Additional experiments were carried out by using confocal microscopy, immmuno-electron microscopy and quantitative morphometry to measure the amount of autophagoproteasomes compared with pure UP or ATG components. All the data reported here were originally generated by the authors of the present manuscript when providing early electron microscopy evidence.

To remark the natural occurrence of autophagoproteasomes even when ATG machinery was depressed a series of experiments was carried out in glioblastoma cell lines (U87MG). In fact these eukariotic cells possess a weak ATG machinery which is due to the up-regulation of mTOR (Jiang et al., 2009; Fan and Weiss, 2012; Arcella et al., 2013; Catalano et al., 2015). In these cells we produced a dose-dependent recovery of ATG activity which shifted from down- to up-regulation under the effects of increasing doses of the mTOR inhibitor rapamycin. This was planned to understand the relationship between autophagoproteasomes and the level of mTOR inhibition/ATG activation.

In fact, if the autophagoproteasome represents a solid reality, then one should expect it to occur even when ATG activity is weak. Again, if the occurrence of autophagoproteasomes is related to ATG activity, then one would expect that recovery of ATG activity under a gold-standard ATG activator (rapamycin) should lead to an increase in the number of autophagoproteasomes.

Since these experiments demonstrate a remarkable merging between ATG and UP components within the very same autophagoproteasomes, further experiments were designed to assess the potential occurrence of molecular binding between UP and ATG components. Therefore, we carried out co-immunoprecipitation experiments, both in baseline conditions and following rapamycin. This inaugural article is aimed to provide functional and morphological evidence for a novel anatomical organelle, which is likely to be key in understanding neuronal (and cell) metabolism and in neurobiology of disease.

## Materials and Methods

### Cell Lines

The human glioblastoma cell lines U87MG (grade-IV), were obtained from Cell Bank (IRCC San Martino-IST, Genova). U87MG were maintained in DMEM medium (Sigma, Italy) supplemented with 10% Fetal bovine serum (FBS; Sigma, Italy), 1% of MEM non-essential amino-acid (MEM-NEAA), penicillin (50 IU/mL), and 100 μg streptomycin. Cell lines were maintained at 37°C in a humidified atmosphere containing 5% CO_2_ and the medium was renewed 2–3 times per week. at 37°C, 5% CO_2_ and 95% of humidity. For transmission electron microscopy (TEM) 1 × 10^6^ cells were seeded in 6-well plates in a final volume of 2 ml/well. For confocal light microscopy 5 × 10^2^ U87MG cells were seeded on cover slips in 24-well plates in a final volume of 1 ml/well while PC12 cells were seeded at a density of 3 × 10^4^ cells in a 24-well plates in culture medium (2 ml/well). After 24 h at 37°C in 5% CO_2_, both cell lines were treated with rapamycin (1, 10, 100 and 1000 nM for 24 h). Dilutions of rapamycin were obtained by a stock solution (1 μM of rapamycin dissolved in culture medium containing 10% DMSO). Control cells were maintained in culture medium containing 0.01% DMSO.

Additional data were produced in rat PC12 cell line to document the species-independency of autophagoproteasome. PC12 Cell line was obtained from the Cell Bank (IRCC San Martino-IST, Genova). The cells were grown in RPMI 1640 medium supplemented with 10% heat-inactivated horse serum, 5% FBS, penicillin (50 IU/mL), and streptomycin (50 mg/mL). Cells were maintained at 37°C in a humidified atmosphere containing 5% CO_2_ and the medium was renewed 2–3 times per week. U87MG and PC12 cells were similarly processed for morphometry.

### Confocal Microscopy

Cells were washed in phosphate-buffered saline (PBS) and fixed with methanol at room temperature for 5 min. Antigen retrieval was performed in 100 mM TrisHCl, 5% urea at 95°C for 10 min. After washing in PBS, cells were permeabilized in 0.2% Triton X-100 for 10 min and then blocked in PBST (PBS + 0.1% Tween-20), supplemented with 1% bovine serum albumin (BSA) and 22.52 mg/ml of glycine, for 30 min. Cells were then incubated overnight at 4°C in 1% BSA in PBST containing 1:50 anti-LC3 antibody (Abcam, Cambridge, UK) and 1:15 anti-P20S (Abcam, Cambridge, UK). After extensive washes in PBST, cells were incubated for 1 h at room temperature with a 1:200 dilution of appropriate fluorophore-conjugated secondary antibodies (goat anti-rabbit Alexa 488 and goat anti-mouse Alexa 594, Molecular Probes, Life Technologies) in 1% BSA in PBST. Cells were then washed in PBS and mounted in Prolong Diamond Antifade Mountant (Molecular Probes, Life Technologies). The analysis was performed using a Leica TCS SP5 confocal laser-scanning microscope (Leica Microsystems, Mannheim, Germany) using a sequential scan procedure. Confocal images were collected every 400 nm intervals through the z-axis of sections by means of 63× oil lenses. Z-stacks of serial optical planes were analyzed using the Multicolor Package software (Leica Microsystems). Negative controls were performed, omitting the primary antibodies.

### Co-Immunoprecipitation Assay

U87MG cells were homogenized at 4°C in ice-cold lysis buffer. One microliter of homogenates was used for protein determinations. Proteins (600 μg) were incubated at 4°C overnight with primary rabbit anti-LC3 antibody (3 μg for each sample; Sigma Aldrich, Milan, Italy). The antibody/antigen complex was pulled out of the sample using protein A-sepharose beads. This process isolated the protein of interest from the rest of the sample. Proteins were separated on sodium dodecyl sulfate polyacrylamide gel (15%) and transferred on Immuno-polyvinylidene difluoride (PVDF) membrane (Biorad, Milan, Italy) for 1 h. Filter was blocked for 1 h in Tween-20 Tris-buffered saline (TTBS; 100 mM Tris-HCl, 0.9% NaCl, 1% Tween 20, pH 7.4) containing 5% non-fat dry milk. Blot was incubated at 4°C overnight with primary antibody mouse monoclonal anti-Proteasome 20S (1:100, Abcam); it was washed three times with TTBS buffer and then incubated for 1 h with secondary peroxidase-coupled antibody (anti-mouse, 1:7000; Calbiochem, Milan, Italy). Then blot was incubated with primary rabbit anti-LC3B antibody (1:6000, Sigma Aldrich) for 1 h at room temperature. Finally, filter was washed three times with TTBS buffer and then it was incubated for 1 h with secondary peroxidase-coupled antibody (anti-rabbit, 1:7000; Calbiochem, Milan, Italy). Immunostaining was revealed by enhanced chemoluminescence (GE Healthcare, Milan, Italy).

### Transmission Electron Microscopy

U87MG cells were centrifuged at 1000 g for 5 min. After removal of the supernatant, pellet was thoroughly rinsed in PBS. Fixation was carried out with a solution containing 2.0% paraformaldehyde/0.1% glutaraldehyde in 0.1 M PBS (pH 7.4) for 90 min at 4°C. This procedure was validated by previous studies as the best fixing procedure for immunocytochemistry when studying autophagy vacuoles. In fact, this fixing solution allows a minimal cover of antigen epitopes preserving the morphology of the tissue. Moreover, this method prevents the occurrence of specific TEM artifacts due to high aldehydes concentrations, which could impair the detection of authentic autophagy vacuoles (Fornai et al., 2001). Specimens were post-fixed in 1% OsO_4_ for 1 h at 4°C; they were dehydrated in ethanol and embedded in Epoxy-resin.

For ultrastructural morphometry, sections were examined directly at TEM at a magnification of 8000×. Each grid contained non-serial sections to count at least 10 cells.

Several grids were observed in order to obtain a total number of at least 50 cells for each experimental group. This number was obtained by sampling cells from at least three experiments. Data obtained in this series of experiments were overlapping those previously measured along the last decade in about 15 pilot studies.

### Details on Post-Embedding Procedure

Specimens were post-fixed in 1% OsO_4_ for 1 h at 4°C and then they were dehydrated in ethanol and embedded in epoxy resin. Ultrathin sections (40–50 nm) of U87MG were cut at ultramicrotome. Similarly to fixing, post-fixing and embedding procedures were validated on pilot studies and they were reported by current literature as optimal conditions for immunogold-based ultrastructural morphometry. We avoided routine method, which consists in avoiding osmium post-fixing and embedding specimens in acrylic resins after fixing with aldehydes only. In fact, such a procedure despite preserving epitopes impairs the preservation of the finest sub-cellular architecture. The method employed here, which combines aldehyde and mild OsO_4_ as first and second fixing steps allows minimal epitope covering while preserving cell architecture and providing an optimal “contrast effect” of various cell compartments. This method allows preserving sub-cellular structures with acceptable epitope integrity (Bendayan and Zollinger, 1983; D’Alessandro et al., 2004).

In fact, osmium enhances the contrast of various cytosolic compartments by marking membranes phospholipids, as clearly confirmed by Swanlund in describing the gold standard of TEM procedures in studying autophagy (Swanlund et al., 2010). Again, the binding of osmium to cell membranes prevents the formation of membranous artifacts, which may mimic ATG vacuoles.

Post-fixed samples were then embedded using epoxy resin. We used epoxy resin, instead of acrylic resin such as LR White, since it is well-established and commonly used as embedding media for TEM, allowing an optimal ultrastructural resolution.

The post-embedding was carried out collecting ultrathin sections on nickel grids and incubating them in aqueous saturated sodium metaperiodate (NaIO_4_) for roughly 30 min at room temperature in order to remove OsO_4_ from the samples. After washing with PBS pH 7.4, ultrathin sections were processed for immunocytochemistry. The NaIO_4_ is an oxidizing agent which attacks the hydrophobic alkane side-chains of epoxy resin (Bendayan and Zollinger, 1983; Causton, 1984) making the sections more hydrophilic and allowing a closer contact between immunogold-conjugated antibodies and the antigens exposed on the surface of each section. The solution enables the detection of specific immunogold placement within a context of subcellular integrity, which allows counting molecules within well-delineated specific cell compartments.

### Immunocytochemistry

Grids were treated with cold PBS containing 10% goat serum and 0.2% saponin to block non-specific antigenic sites for 20 min at room temperature.

Following the blocking step, samples were incubated with two primary antibodies in order to obtain a co-localization (either with LC3 + P20S, LC3 + beclin-1, LC3 + 19S; P20S + beclin-1; P20S + 19S). The different primary antibodies were: anti-LC3 (Abcam, Cambridge, UK, diluited 1:50); anti-beclin-1 (Abcam, Cambridge, UK, diluited 1:50); anti-P20S (Abcam, Cambridge, UK, diluited 1:10); anti-PA700 (19S/PA700 Chemicon, Temecula, CA, USA). Antibodies against 19S proteasome subunit and the ATG marker beclin-1 were used only for validating the co-localization of ATG and UP with autophagoproteasomes. Only LC3 and P20S antibodies were used for counts at ultrastructural morphometry. Antibody specificity was assessed by a number of studies which were partially reported in Table 1 (extramural evidence) and they were routinely used for at least 10 years in our lab (intramural evidence). The marker specificity meant as the subcellular structures which were the object of this study (vacuoles) was assessed in full agreement with the third edition of the Guidelines for the use and interpretation of assays for monitoring autophagy (Klionsky et al., 2016).

**Table 1:** **Sources and references for all the antibodies reported in the present study**.

Antibodies	References
LC3 (Nanotools)	Ladoire et al. (2012), Hiniker et al. (2013), Martinet et al. (2013), Koukourakis et al. (2015)
LC3 (Santa Cruz)	Balgi et al. (2009), Hu et al. (2012), Chen et al. (2013), Lisiak et al. (2014)
LC3 (Abcam)	Porter et al. (2013), Chen et al. (2014b), Pla et al. (2014), Wang et al. (2014)
LC3 (Sigma)	Klionsky and Emr (2000), Klionsky et al. (2003), Kuma et al. (2004)
Proteasome 20S (Abcam)	Hendil et al. (1995), Kovarik et al. (2010), Vilchez et al. (2012), Stout et al. (2013)
Proteasome 20S (Chemicon)	Castino et al. (2008)
Beclin I (Abcam)	Chen et al. (2012, 2014a), Abdulrahman et al. (2013)
Beclin I (Santa Cruz)	Lenzi et al. (2012), Guo et al. (2015)
19S/PA700 (Abcam)	Sun et al. (2005), Rousseau et al. (2009), Zhou et al. (2011)
19S/PA700 (Chemicon)	Castino et al. (2008)

Incubations were carried out in ice cold PBS containing 1% goat serum and 0.2% saponin in a humidified chamber overnight at 4°C.

Ultrathin sections were washed in cold PBS, they were incubated with gold-conjugated secondary antibodies (10 nm gold particles anti-LC3; 20 nm gold particles anti-P20S, BB International), and they were diluted 1:20 in PBS containing 1% goat serum and 0.2% saponin for 1 h, at room temperature.

After rising in PBS, grids were incubated with 1% glutaraldehyde for 3 min, they were washed in distilled water (to remove trace of salts and preventing precipitation of uranyl acetate), and they contrasted with uranyl acetate (saturated solution in distilled water) and lead citrate to be finally observed by using a Jeol JEM SX 100 electron microscope (Jeol). Control sections were obtained by omitting the primary antibody and by incubating with the secondary antibody only.

### Assessment of Autophagy-Like Vacuoles, LC3 Positive, and/or P20S Positive Vacuoles

Pellets from U87MG cell lines allow an appropriate condition for quantitative morphometry since ultrathin sections contain cells randomly oriented owing a similar chance to be selected. In fact, counts obtained from different cell pellets are very consistent as witnessed by the small standard error (Ylä-Anttila et al., 2009; Lenzi et al., 2012).

Ultrastructural morphometry was carried out at 8000× magnification in order to distinguish autophagy (ATG)-like vacuoles and LC3 or P20S vacuoles and LC3 + P20S positive vacuoles. Each grid contained non-serial sections, and for each grid we observed an average of 10 cells. Several grids were observed in order to obtain a total number of 50 cells from each experimental group.

We scored as ATG-like vacuoles both, double or multiple membranes (autophagosomes-like) containing cytoplasmic material and electrondense membranous structures according to previous studies (Fornai et al., 2007; Lenzi et al., 2012). We counted the number of ATG-like vacuoles for each cell, and then we calculated the mean value per cell. Authentic ATG vacuoles were instead defined by the occurrence of LC3 with an ATG-like vacuole. Moreover, we calculated the percentage of LC3 only, P20S only and LC3 + P20S positive vacuoles.

### Count of Immunogold Particles

Measurement of immunogold particles (10 nm or 20 nm) placed in the cytosol, or within ATG vacuoles was carried out by electron microscopy at 8000× magnification according to Lucocq et al. (2004). In fact, 8000× corresponds to the minimal magnification at which gold particles and cell compartments can be still identified concomitantly with whole organelles and big cell compartments.

Grid squares containing labeled cells were chosen randomly to start the count. In particular, starting at a grid square corner, the whole of the sectioned pellet within that grid square was scanned continuously in equally spaced parallel sweeps across the specimens. Random selection makes the quality of the scanning independent either from cell density or intensity of gold labeling. For LC3 and P20S, in each cell we counted the number of anti-LC3 or anti-P20S immunogold particles placed in the cytoplasm, and we expressed the mean value of immunogold particles for cell out of 50 cell counted. Moreover, we counted the number of anti-LC3 or anti-P20S immunogold particles within ATG-like vacuoles (to identify autophagosomes and autophagoproteasomes as reported above). For co-localization, we counted the ATG-like vacuoles that possessed both immunogold particles (10 nm for LC3 and 20 nm for P20S).

### Statistics

For confocal microscopy the amount of P20S + LC3 puncta were counted. Values were expressed as the mean number of puncta per cell counted in two separate experiments each one carried out in duplicate. We counted 50 cells for each group.

For ultrastructural morphometry values were expressed using the absolute value: (i) ATG-like vacuoles; (ii) cytosolic LC3 or P20S immunogold particles; (iii) LC3 or P20S-positive vacuoles; (iv) P20S + LC3-positive vacuoles; or as percentage of normal numerical distributions: (i) LC3 or P20S and P20S + LC3 positive vacuoles out of total ATG-like vacuoles; (ii) the number of LC3 or P20S immunogold particles within vacuoles out of total immunogold particles of LC3 or P20S respectively; (iii) P20S + LC3 positive vacuoles out of the single positive vacuoles (LC3 solely and P20S solely, respectively). Moreover, values for ultrastructural morphometry were also expressed as a ratio as follows: (i) cytosolic LC3 or P20S particles out of total ATG-like vacuoles; (ii) LC3-positive vacuoles out of total P20S-positive vacuoles and the reciprocal value; (iii) cytosolic P20S particles out of cytosolic LC3 particles; (iv) P20S particles within vacuoles out of cytosolic P20S particles. These data were obtained in a total of 50 cells per group which were randomly harvested from three separate experiments. Indeed, the high consistency of each value made the randomization useless since the mean obtained for each value from each group were overlapping the values we constantly measured during the past decades in pilot non-systematic experiments. Nonetheless, in the recent data collection we wish to systematically establish the mild variability of these biological phenomena which led to S.E.M often ranging around 5% of the mean.

Data are reported as the mean or the mean percentage ± S.E.M.

Inferential statistics to compare groups was carried out by using one-way analysis of variance, ANOVA, with Sheffè’s *post hoc* analysis (H_0_, null hypothesis, was rejected for *P* ≤ 0.05).

## Results

### Ultrastructural Studies

#### Plain Transmission Electron Microscopy of Autophagy-Like Vacuoles

**Figure 1:**
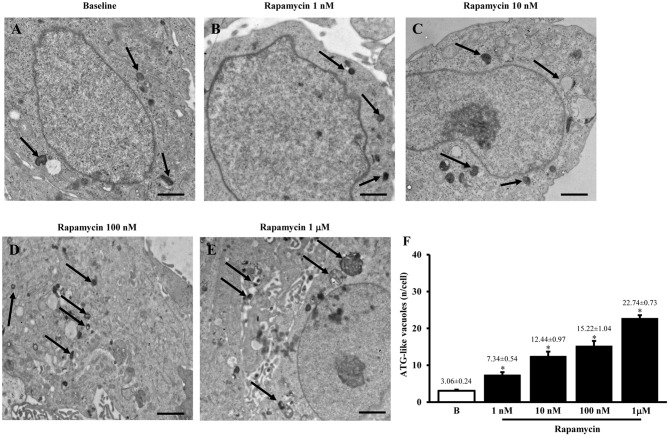
**Plain electron microscopy of U87MG cells**. Representative pictures of ATG-like vacuoles in the cytoplasm in baseline conditions (arrows)** (A)** and after increasing doses of rapamycin **(B–E)** as visualized in representative micrographs. The mean number of ATG-like vacuoles per cell was counted and values were reported in graph **(F)**. This graph shows that rapamycin induces dose-dependently (1 nM; 10 nM; 100 nM and 1 μM) an increase in the number of ATG-like vacuoles compared with baseline conditions **(B)**. Values are given as the mean ± S.E.M. Comparisons between groups were made by using one-way ANOVA. *P ≤ 0.05 compared with baseline conditions. Scale bars **(A–E)** = 1μm.

The typical ultrastructure of U87MG cells in baseline conditions is represented in Figure 1 showing depressed autophagy activity as expected from mTOR up-regulation, which occurs in this cell line, which in turn produces only a few ATG-like vacuoles. Accordingly, as shown in representative pictures reported in Figures 1A–E and in the graph of Figure 1F the amount of ATG-like vacuoles which was counted by using plain electron microscopy dose-dependently increases under the effects of the mTOR inhibitor rapamycin (Figures 1D,E compared with Figure 1A). In detail, the number of vacuoles dramatically increased following the highest dose of rapamycin (1 μM), which leads to a roughly 7-fold increase. The consistency of these phenomena is witnessed by the low variability in the mean obtained from 50 cells from each group as it appears from the S.E.M. of each value (Figure 1F).

#### LC3 Immunocytochemistry

**Figure 2:**
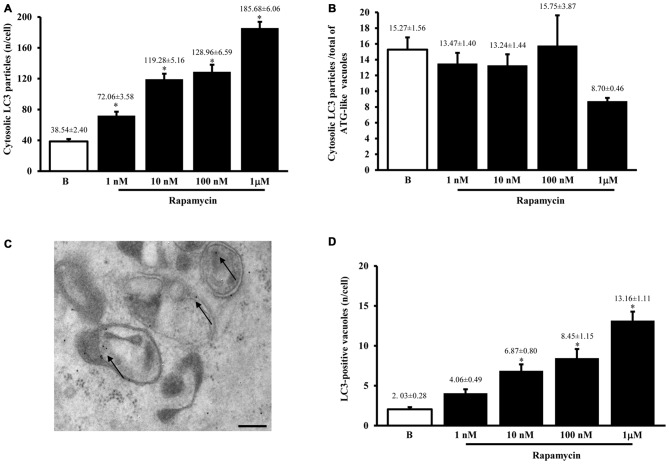
**LC3 immunocytochemistry**. The number of cytosolic LC3 immunogold particles significantly increases dose-dependently after rapamycin (1; 10; 100; nM and 1 μM) compared with baseline conditions **(B)** as reported in graph **(A)**. When rapamycin-dependent increase in immunogold particles was plotted vs. rapamycin-dependent increase in ATG-like vacuoles reported in Figure 1F, we measured a similar trend as witnessed by the steady values reported in graph **(B)** for baseline conditions **(B)** and different doses of rapamycin (1 nM; 10 nM; 100 nM and 1 μM). This suggests that LC3 particles and ATG-like vacuoles express the same phenomenon (ATG status). To confirm this analogy we carried out LC3 immunocytochemistry for ATG vacuoles as shown in representative picture **(C)** where authentic ATG vacuoles possessing different shapes and a double membrane were stained with LC3 immunogold particles (shown by arrows; gold standard procedure according to Klionsky et al., 2016). This procedure allowed to count authentic ATG LC3-positive vacuoles, which were reported in graph **(D)**. This graph shows that rapamycin induces dose-dependently (1 nM; 10 nM; 100 nM and 1 μM) an increase in the number of ATG LC3-positive vacuoles compared with baseline conditions **(B)**. The increase in ATG-like vacuoles observed in Figure 1F was matched by such an increase in ATG LC3-positive vacuoles reported in panel **D**. In **(C)** vacuoles stained with LC3 immunogold particles (arrows) show different shape. The absolute number of LC3-positive vacuoles increases dose dependently **(D)**. Values are given as the mean ± S.E.M. Comparisons between groups were made by using one-way ANOVA. *P ≤ 0.05 compared with baseline conditions. Scale bar **(C)** = 250 nm.

**Figure 3:**
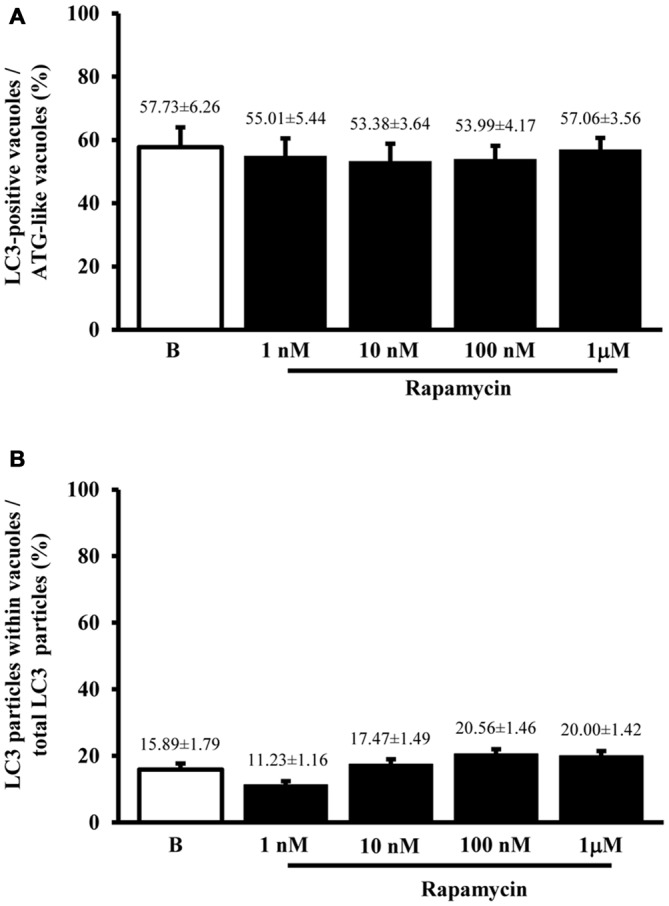
**Comparisons between LC3 immunostaining and ATG vacuoles**. As hypothesized from the similarities between graphs Figures 1F,D, when we plotted the mean number per cell of LC3 positive vacuoles vs. the mean number per cell of ATG-like vacuoles expressed as percentage **(A)** we failed to observe any significant difference for different doses of rapamycin (1 nM; 10 nM; 100 nM and 1 μM) and baseline conditions **(B)**. This confirms the same nature of both phenomena (ATG status). In fact, the values remained steady also in graph **(B)** when the dose-dependent increase in the number of LC3 particles within vacuoles was plotted vs. the dose-dependent increase in the number of total LC3 particles for increasing doses of rapamycin (1 nM; 10 nM; 100 nM and 1 μM) and baseline conditions **(B)**. Thus, graph **(B)** confirms that LC3 increase expresses the increase in authentic ATG vacuoles. Values are given as the mean percentage ± S.E.M. Comparisons between groups were made by using one-way ANOVA.

When measured at high magnification counts of immunogold staining it confirmed low baseline ATG activity as indicated by depressed levels of the ATG hallmark protein LC3 (Figure 2A). This effect was widespread in the cell, independently from which specific compartment was analyzed (ATG-like vacuoles, mitochondria or other structures). In keeping with the increase in ATG-like vacuoles counted in Figure 1F, we documented a dramatic amount in cytosolic LC3 immunogold particles under the effects of rapamycin (Figure 2A). Remarkably, such an increase was dose-dependent, similarly to that measured for ATG-like vacuoles for the highest dose of rapamycin (185.68 ± 6.06 compared with 38.54 ± 2.40 for baseline conditions, Figure 2A). The analogy between these effects was confirmed by counting the ratio between LC3 immunogold particles and ATG-like vacuoles in baseline conditions and following increasing doses of rapamycin. These counts are reported in Figure 2B, which demonstrates that the ratio remains steady in baseline conditions and following increasing doses of rapamycin. This suggests that both the widespread increase of LC3 we measured in the cytosol and the increase of ATG-like vacuoles depend on the same phenomenon relying on rapamycin-induced mTOR-inhibition, which in turn, produces ATG stimulation. In fact, when counting the number of authentic LC3-positive ATG vacuoles (which represent the gold standard structures to assess the autophagy status, see representative Figure 2C) we could document the increase in ATG activity induced by rapamycin, which produced a significant increase in the absolute number of LC3-positive ATG vacuoles (Figure 2D). Again, the magnitude of such an increase overlaps with that measured for ATG-like vacuole and for LC3 cytosolic particles as previously reported (Figures 1F, 2A, respectively). In order to further validate the measurement of LC3 cytosolic particles and ATG-like vacuoles we measured the percentage of LC3 positive vacuoles out of total ATG-like vacuoles. This measurement led to a roughly half of authentic (LC3 positive) ATG vacuoles out of total ATG-like vacuoles. Remarkably, such a percentage was not modified by ATG activation and it remained steady along increasing doses of rapamycin (Figure 3A). Again, when the percentage of LC3 particles within vacuoles was counted out of total LC3 particles we detected steady values (Figure 3B). These latter data indicate that the vacuoles we counted as ATG-like are indeed roughly double of authentic (LC3 positive) ATG vacuoles. It is likely that ATG-like vacuoles do correspond to authentic ATG vacuoles since they are similarly depressed in baseline conditions and they similarly increase under rapamycin administration. Thus, the issue of defining ATG vacuoles as ATG-like is rather methodological. In fact, it is likely that the thickness of the ultrathin electron microscopy sections which is roughly a half of the diameter of an ATG vacuole leads to a 50% chance to detect LC3 particles within the vacuoles which are likely to be, in any case, authentic ATG structures. This concept is in line with what recently expressed by Lucocq and Hacker (2013).

#### P20S Immunocytochemistry

**Figure 4:**
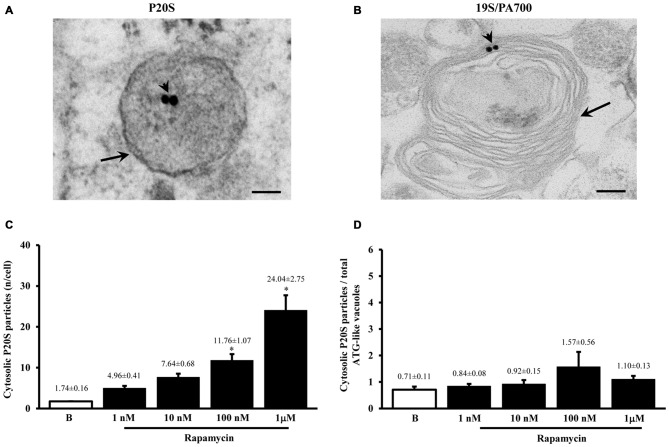
**Ultrastrucure of ubiquitin proteasome (UP) components and their presence within ATG-like vacuoles**. UP components were identified by immunocytochemistry against either P20S or 19S/PA700 as shown in representative micrograph **(A)** where arrowheads show P20S-bound immunogold particles and representative micrograph **(B)** where arrowheads show 19S/PA700-bound immunogold particles. In both **(A,B)** these proteasome components are shown in the context of multiple membranes ATG-like vacuoles shown by arrows. However, the presence of UP component also occurred in the cytosol. In fact, as measured in graph **(C)**, we carried out an extensive count of total cytosolic UP component. For these counts we used P20S immunogold particles. Again, even in this case we measured a dose-dependent increase in the number of P20S particles for increasing doses of rapamycin (1 nM; 10 nM; 100 nM and 1 μM) number of ATG-like compared with baseline conditions **(B,C)**. When we compared such a rapamycin-dependent increase in cytosolic UP components with the dose-dependent increase in ATG-like vacuoles primarily expressed by graph Figure 1F we obtain graph **(D)** showing that the ratio between P20S immunogold particles out of total ATG-like vacuoles was steady for increasing doses of rapamycin (1 nM; 10 nM; 100 nM and 1 μM) number of ATG-like compared with baseline conditions **(B,D)**. Values are given as the mean ± S.E.M. Comparisons between groups were made by using one-way ANOVA. *P ≤ 0.05 compared with baseline condition. Scale bars **(A,B)** = 112 nm.

**Figure 5:**
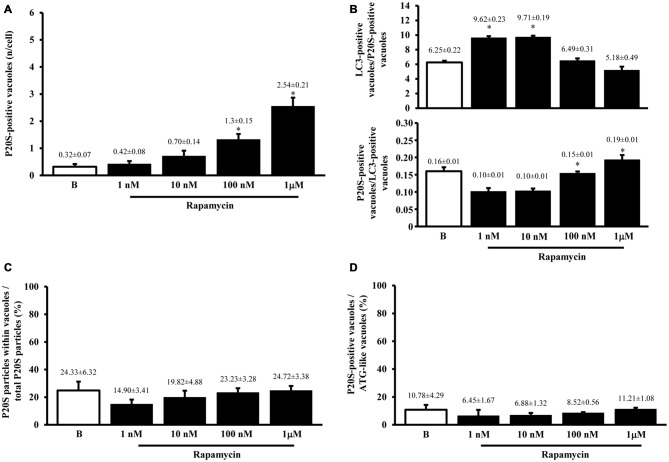
**Significance of increases in P20S compared with LC3 and ATG vacuoles**. As expected from graph Figure 4D, the number of P20S-positive vacuoles increases dose-dependently for increasing doses of rapamycin (1 nM; 10 nM; 100 nM and 1 μM) compared with baseline conditions **(A,B)**. Such an increase possessed the same trend of those observed either for LC3-immunogold or for ATG vacuoles. However, slight though significant differences can be detected. In fact, when plotting the number of LC3 on P20S positive vacuoles or vice versa (upper and lower graph of **B**), we found a significant prevalence of LC3 over P20S for lower rapamycin doses which was reciprocated by a prevalence of P20S over LC3 for higher rapamycin doses (**B** upper and lower graph, respectively). This subtle difference in the dose-dependent increase in components belonging to different clearing systems indicates that mTOR inhibition by rapamycin is expected to produce dose-dependent different stimulations of ATG and UP. This difference should not be misleading since in both cases both systems are overexpressed. Despite a total increase, when the amount of P20S-positive vacuoles was plotted vs. the number of total P20 particles the percentage values for each dose of rapamycin and baseline conditions remained steady **(C)** apart from a slight decrease for the lowest dose of rapamycin. Again, confirming the similar nature of P20S-positive vacuoles with ATG components, we measured the same percentage of P20S-positive vacuoles out of the total ATG-like vacuoles following different doses of rapamycin (1 nM; 10 nM; 100 nM and 1 μM) and baseline conditions **(B,D)**. Values are given as the mean or percentage of the mean ± S.E.M. Comparisons between groups were made by using one-way ANOVA. **(A)** *P ≤ 0.05 compared with baseline condition. **(B)** *P ≤ 0.05 compared with the other groups.

Despite the vacuoles counted at plain TEM or following immunostaining correspond to ATG organelles, in the past decades, during various experiments, we noticed that proteasome components could be detected at this level. Therefore, in line with the purpose of the study, we detailed the occurrence of UP components within these ATG vacuoles. As shown in representative Figures 4A,B we confirmed the presence of both P20S and 19S (PA700), respectively, within ATG-like vacuoles. Thus, as it was carried for LC3 (Figure 2A) we counted the amount of P20S-immunogold particles in baseline conditions and under ATG stimulation (i.e., rapamycin). Remarkably, despite the absolute number of P20S in baseline conditions was lower than LC3 (1.74 ± 0.16 compared with 38.54 ± 2.40, respectively), the increase we counted in P20S particles was remarkable (24.04 ± 2.75, at 1 μM rapamycin compared with 1.74 ± 0.16, in baseline conditions, Figure 4C). Similarly to LC3, despite both P20S particles and ATG-like vacuoles increased significantly under the effects of rapamycin their ratio stays steady (Figure 4D). Remarkably, when we counted the number of P20S positive vacuoles we found that although they were present in baseline conditions, there was a dose-dependent increase in P20S positive vacuoles occurring following rapamycin (Figure 5A). This increase was reminiscent of that occurring for LC3 as reported in Figure 2D. In fact, when the ratio between LC3 positive vacuoles and P20S positive vacuoles (and vice versa, two reciprocal graphs of Figure 5B) was plotted in baseline conditions and following different doses of rapamycin values were quite steady (Figure 5B), despite a slight increase of LC3 over P20 vacuoles at lower rapamycin doses and the opposite trend for the highest doses of rapamycin (see reciprocal graphs of Figure 5B). This slight effect suggests a prevalence of ATG vs. UP for low mTOR inhibition with a shift towards a more UP-based clearance for higher extent of mTOR inhibition. Of course these data are merely based on protein amount and need further studies to confirm whether this translates into enzymatic activity.

Altogether graphs Figures 5A,B indicate that P20S just like LC3 is increased by rapamycin within ATG vacuoles. Thus, both ATG and UP markers, associate significantly with ATG vacuoles. This is further confirmed by graph Figure 5C showing that the ratio between the dose-dependent increase in P20S particles within vacuoles and the increase in the total number of cytosolic P20S particles similarly results in steady values (Figure 5C). This latter graph overlaps again with what reported for LC3 particles in Figure 3B. This means that neither LC3 (Figure 3B), nor P20S (Figure 5C), vary their percentage of compartmentalization within vacuoles following increasing doses of rapamycin. It is remarkable that despite the net amount of P20S is way lower compared with LC3 (Figures 2A, 4C) the percentage of compartmentalization of these molecules within ATG-like vacuoles is similar for LC3 (Figure 3B) and P20S (Figure 5C) in baseline conditions and after different doses of rapamycin. This indicates that P20S is related with ATG vacuoles as much as LC3. Similarly to LC3, the percentage of P20S immunopositive vacuoles was not modified following rapamycin (Figure 5D).

**Figure 6:**
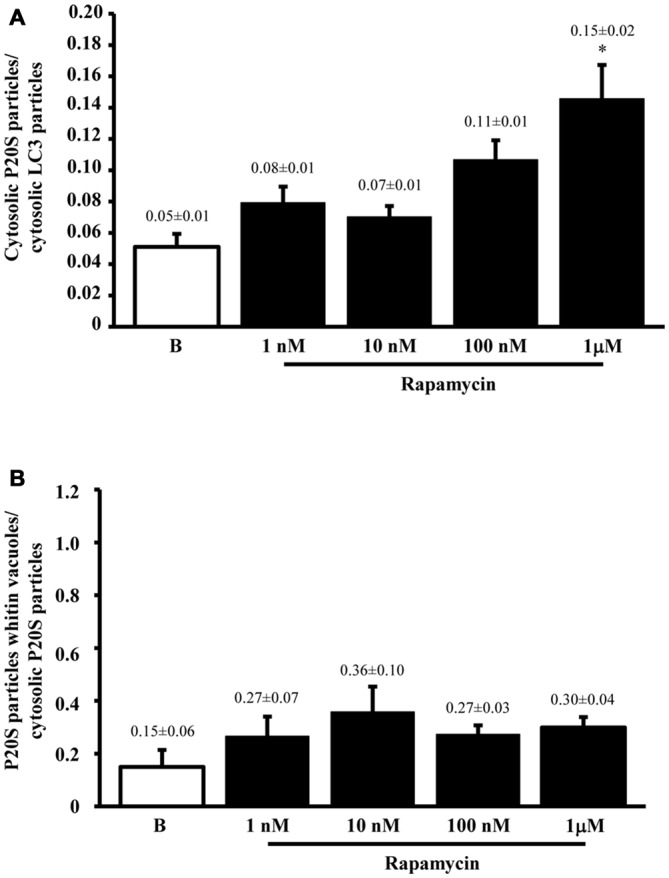
**P20S-positive compared with LC3-positive particles and P20-positive vacuoles**. Graph **(A)** confirms a more pronounced increase in UP vs. ATG components (P20S compared with LC3) for the highest dose of rapamycin. In fact, both P20S and LC3 particles increase dose-dependently for increasing doses of rapamycin (1 nM; 10 nM; 100 nM and 1 μM) compared with baseline conditions **(B)** but the highest dose of rapamycin which produced a significantly higher increase for P20S compared with LC3. The ratio between compartmentalized P20S within vacuoles out of total cytosolic P20S shows a steady trend towards higher values following rapamycin compared with baseline **(B)**. Values are given as the mean ± S.E.M. Comparisons between groups were made by using one-way ANOVA. *P ≤ 0.05 compared with baseline condition.

The results of ultrastructural morphometry carried out so far, distinctly for LC3 and P20S, demonstrate a number of similarities for these two markers in relationship with ATG vacuoles. In fact, both markers respond similarly to dose-dependent inhibition of mTOR. Nonetheless, the occurrence of LC3 is much abundant than P20S in baseline condition which makes the percentage of LC3 positive vacuoles higher than P20S positive vacuoles. However, this difference is significantly attenuated by the highest dose of rapamycin. This suggests that LC3 and P20S similarly increase following maximal mTOR inhibition however the polarization of their placement towards ATG vacuoles is higher for P20S compared with LC3. This makes LC3 a more abundant and specific marker for ATG vacuoles but it indicates that the placement of P20S within ATG vacuoles is more sensitive to the amount of mTOR inhibition. In fact, when we measured the ratio between the amount of cytosolic P20S over cytosolic LC3 in baseline conditions and following the highest dose of rapamycin, we found that the increase in P20S particles significantly exceeded the increase in LC3 particles (Figure 6A). The significance of this finding is further magnified by considering that the ratio between compartmentalized P20S within vacuoles over dispersed cytosolic P20S increased two-fold for the highest dose of rapamycin (Figure 6B).

#### P20S + LC3 Immunocytochemistry

**Figure 7:**
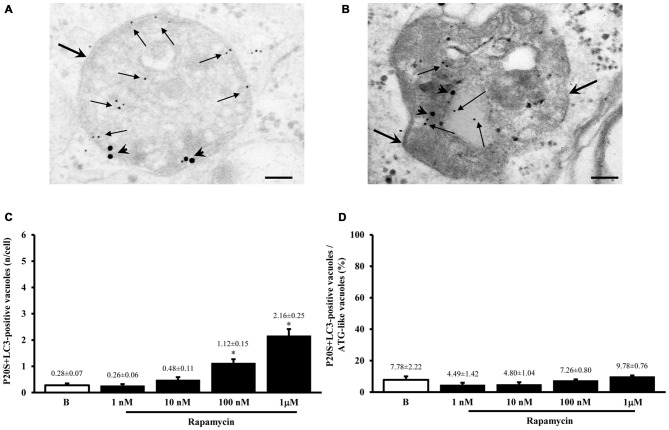
**Occurrence of autophagoproteasome**. When LC3 and P20S immunogold staining was carried out concomitantly the occurrence of both P20S particles and LC3 particles within the very same ATG vacuoles was documented as shown in representative micrograph **(A)**. In this picture P20S staining is represented by 20 nm immunogold particles evidenced by arrowheads, while LC3 staining is represented by smaller (10 nm) immunogold particles evidenced by full thin arrows. To further validate co-localization of UP with ATG components P20S staining was also combined with another specific marker of ATG which is Beclin-1 **(B)**. In both micrographs **(A,B)** thick arrows point to double membranes limiting vacuoles. The co-localization of P20S immunogold particles (20 nm; arrowheads) with Beclin-1 immunogold particles (10 nm, arrows) within a vacuole confirms the existence of a merging organelle named autophagoproteasome. When the mean number of autophagoproteasomes occurring in the cell was counted in graph **(C)** a dose-dependent increase of P20S + LC3 positive vacuoles after rapamycin (1 nM; 10 nM; 100 nM and 1 μM) compared with baseline conditions **(B)** was documented. In order to correlate the occurrence of autophagoproteasome with the amount of the primary ATG-like vacuoles measured in Figure 1F, we calculated the percentage of LC3 + P20S positive vacuoles out of total ATG-like vacuoles **(D)**. Remarkably, such percentage remains steady following increasing dose of rapamycin (1 nM; 10 nM; 100 nM and 1 μM) compared with baseline conditions **(B)**. Values are given as the mean or the percentage of the mean ± S.E.M. Comparisons between groups were made by using one-way ANOVA. *P ≤ 0.05 compared with baseline condition. Scale bars **(A)** = 140 nm; **(B)** = 118 nm.

**Figure 8:**
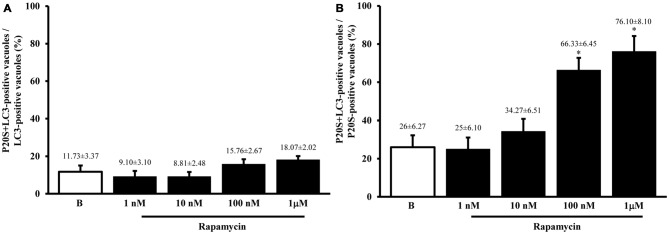
**The occurrence of autophagoproteasomes compared with either LC3- or P20S-positive vacuoles**. In order to compare the occurrence of the autophagoproteasome with the occurrence of total vacuoles positive for LC3 or P20S we measured the ratio between P20S + LC3 positive vacuoles and LC3 expressed as percentage graph **(A)** or the ratio between P20S + LC3 positive vacuoles and P20S expressed as percentage graph **(B)**. These measurements were carried out after rapamycin (1 nM; 10 nM; 100 nM and 1 μM) compared with baseline conditions **(B)**. Remarkably, when the percentage of autophagoproteasome was calculated out of total LC3-positive vacuoles the graph shows steady values; in contrast, when the amount of autophagoproteasomes was plotted against P20S-positive vacuoles, we noticed a significant augmentation in autophagoproteasomes increase which is likely to depend on the trend of the few P20S particles to be dragged by the abundant LC3 molecules to localize within the same compartment under the increasing mTOR inhibition produced dose-dependently by rapamycin **(B)**. For the highest dose of rapamycin most of P20S positive vacuoles are both LC3 and P20S positive which suggests that P20S vacuoles almost correspond to LC3 + P20S positive vacuoles when mTOR is strongly inhibited. Values are given as the mean or the percentage of the mean ± S.E.M. Comparisons between groups were made by using one-way ANOVA. *P ≤ 0.05 compared with baseline condition.

As shown in representative Figures 7A,B, the P20S proteasome marker co-localizes with ATG markers such as LC3 (Figure 7A) and beclin-1 (Figure 7B). When considering the gold standard marker LC3 for quantitative morphometry, we could demonstrate that a few percentage of what was once defined the classic ATG vacuole (LC3 positive) may also stain for the proteasome marker P20S. This co-localization generates a novel entity which corresponds to the autophagoproteasome. Remarkably, in the present study, when counting this double immunostaining we found that the amount of autophagoproteasomes reported in graph Figure 7C is similar to the amount of P20S positive vacuoles counted in Figure 5A. This indicates that P20S positive vacuoles are mostly LC3 + P20S positive autophagoproteasomes. The occurrence of these double clearing vacuoles is lower compared with classic LC3 positive autophagosomes. This is in line with the lower amount of P20S in the cell (both in the cytosol and within autophagy vacuoles) compared with LC3. However, the amount of P20S positive vacuoles and LC3 + P20S positive vacuoles are almost overlapping which suggests that autophagoproteasomes are well defined by the presence of P20S within an LC3 positive vacuole. Interestingly, despite the net increase in all vacuoles (both LC3 positive autophagosomes and LC3 + P20S positive autophagoproteasomes) under the effects of increasing doses of rapamycin, the percentage of LC3 + P20S out of total ATG vacuoles (Figure 7D) and out of LC3 positive ATG vacuoles (Figure 8A) stay quite steady, despite a slight increase. This indicates that increasing mTOR inhibition produces a quite harmonic expression of both clearing pathways leaving intact the ratio between autophagosome and autophagoproteasomes. It is remarkable though, that for the highest dose of rapamycin there was a two-fold increase in autophagoproteasome compared with sole P20 positive vacuoles (Figure 8B). This latter finding suggests that co-localization of P20S within LC3 positive vacuoles prevails for the highest doses of rapamycin. This would be in line with the fact that pure proteasomal degradation is unlikely under powerful ATG stimulation which appears to improve a combined ATG + UP clearing organelle instead.

**Figure 9:**
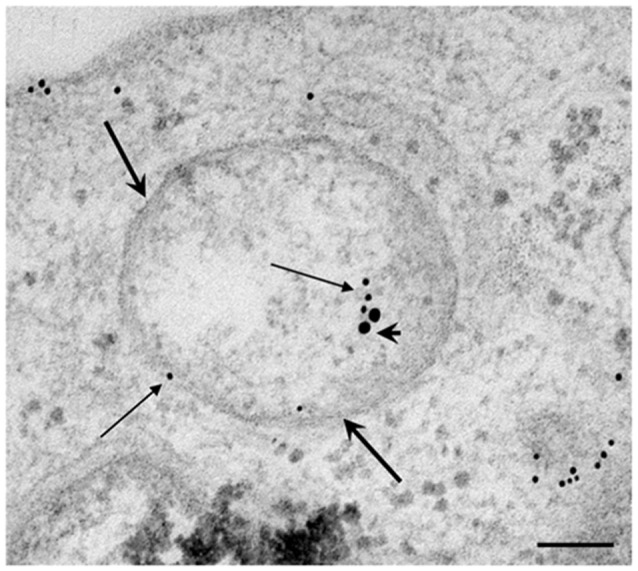
**Electron microscopy of autophagoproteasome in PC12 cells**. The concomitant occurrence of P20S and LC3 immunostainig within a double membrane (thick arrows) vacuole is shown in representative micrograph. The LC3 staining is shown by small (10 nm) immunogold particles indicated by thin arrows while P20S staining is shown by large (20 nm) immunogold particles indicated by arrowheads. This confirms in a rat-derived cell line convergence between autophagy and proteasome in the novel clearing organelle autophagoproteasome. Scale bars = 75 nm.

In Figure 9 we report the co-localization of P20S and LC3 in PC12 cells as shown in representative picture. The amount of autophagoproteasome we counted in ultrathin sections in this rat-derived cell line is 0.26 ± 0.06. This data need to be corrected based on cell surface. Since the surface of PC12 cells is roughly a half of U87MG, we measured a density of autophagoproteasome in PC12 which is two-fold the density counted in U87MG.

### Confocal Microscopy

**Figure 10:**
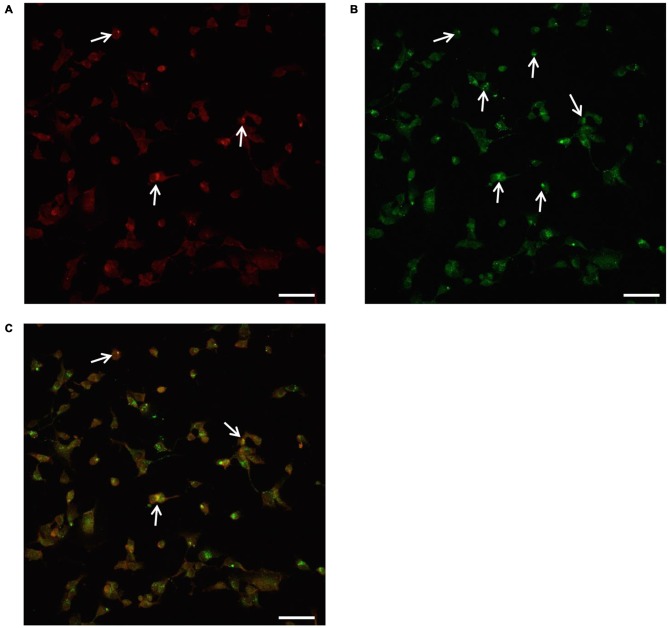
**Confocal microscopy of autophagoproteasomes low magnification**. Confocal microscopy confirms the occurrence of autophagoprotesome as shown by representative images at low magnification. At the highest dose of rapamycin the amount of P20S immunostaining **(A)** is scattered in the cell and only a few red puncta were observed (arrows). At this magnification the difference between P20S immunostaining and the intense LC3 immunostaining as shown by green puncta (arrows) is even more evident **(B)**. Co-localization of P20S + LC3 within the same cells was shown by merging as orange/yellow puncta (arrows) **(C)**. Again these representative pictures at low magnification indicate that, for the highest doses of rapamycin, P20S is mostly dragged down within LC3 positive vacuoles making it more compartmentalized than LC3 itself. Scale bar = 12 μm.

**Figure 11:**
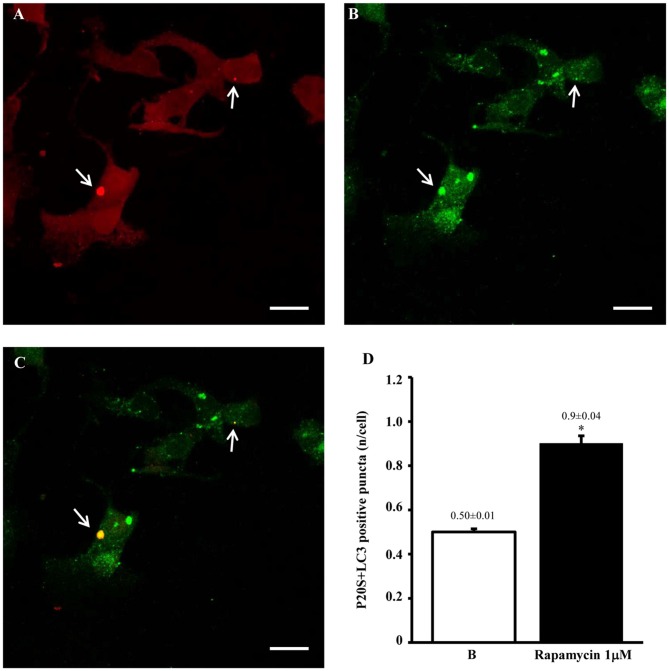
**Confocal microscopy of autophagoproteasomes low magnification**. When confocal microscopy was observed at higher magnification the occurrence of autophagoprotesomes was more evident. In fact, as shown in representative image **(A)** corresponding to the highest dose of rapamycin the amount of P20S immunostaining is quite rare in the cell as shown by arrows marking only a few red puncta. This markedly contrasts with the intense blossoming of LC3 immunostaining showed in **(B)** where several green puncta are marked by arrows. In these cells single puncta are no longer distinguishable due to the spreading of intense immunofluorescence. Remarkably, as suggested by graph Figure 8B and already evident at lower magnification in Figure 10, the very same puncta which were labeled for P20S only **(A)** also stained for LC3 **(C)**. Co-localization of P20S + LC3 is shown by merging orange/yellow puncta (arrows). These merging structures do represent autophagoproteasomes. The amount autophagoproteasomes under the pressure of strong mTOR inhibition (causing ATG activation) corresponds quite exactly to the amount of P20S-positive vacuoles since they are the same structures. In this way, representative images (Figures 10, 11) provide a “one-glance evidence” of the numerical similarity between autophagoprotesomes and P20S positive vacuoles following 1 μM rapamycin which was measured in graph Figure 8B. Again this suggests that, for the highest doses of rapamycin P20S is selectively dragged down within LC3 positive vacuoles making it more compartmentalized than LC3 itself. This calls for testing whether potential molecular binding exists between these components. Confirming again what counted in graph Figure 8B, when we measured merging puncta, in graph **(D)** we found that, for the highest dose of rapamycin, merged puncta increased two-fold compared with baseline **(B)**. In fact, graph **(D)** represents the number of P20S + LC3 positive puncta per cell. Values are given as the mean ± S.E.M. Comparisons between groups were made by using one-way ANOVA. *P ≤ 0.05 compared with baseline conditions. Scale bar = 20 μm.

In order to provide further evidence of merging between ATG and UP components we carried out a few representative confocal microscopy experiments to document merging between ATG and UP reported in representative Figures 10A–C showing at low magnification the occurrence of P20S and LC3 immunostaining which merge at the level of autophagoproteasomes. When compared with TEM micrograph, the occurrence of P20S immunostaining is even more polarized towards LC3 immunopositive area. In contrast, LC3 immunostaining possess a significant amount of single (non-merging) staining. This is emphasized when analyzing confocal images at higher magnification as shown in Figures 11A–C. At high magnification confocal microscopy P20S and LC3 immunostaining co-localize in U87 cells. In Figure 11C a representative image of merging at confocal microscopy provides a rough visualization of an autophagoproteasome despite that no specific cell compartment can be identified. In graph of Figure 11D the mean number of autophagoproteasomes per cell is reported in baseline conditions, which provide quantitative measurement of what could be already appreciated at low magnification in Figure 10. When cells are exposed to the mTOR inhibitor rapamycin at the dose of 1 μM there is a significant increase in the number of autophagoproteasomes-like structures which increase roughly two-fold compared with those counted in baseline conditions. Remarkably, this corresponds to the two-fold increase in autophagoproteasomes of Figure 8B. Due to a lower resolution of confocal compared with TEM, there is an underestimation in the number of autophagoproteasomes, which was calculated using confocal microscopy compared with immuno-electron microscopy (see “Discussion” Section). For the same reason, the amount of immunopositive puncta both in baseline and stimulated conditions is way lower compared with immunogold particles counted at electron microscopy. This discrepancy likely relies on the sensitivity of the methods as reported in the “Discussion” Section. When counted at TEM, the amount of P20S positive vacuoles is comparable with the amount of P20S + LC3 positive vacuoles, which corresponds exactly to the overlapping between P20S positive puncta and merging (P20S + LC3) positive puncta observed at confocal microscopy. However, when observed at TEM, immunogold P20S positive particles exceeds the number of P20S particles within vacuoles. This indicates that at TEM all P20S particles are stochietrically detected, whereas at confocal microscopy this occurs only when P20S is present within vacuoles.

At confocal microscopy a quantitative measurement of LC3 immunopositive puncta was technically impossible due to a spreading of immunofluorescence from the great amount of sources (LC3 immunofluorescent bodies) which contrasts with the clear-cut localization and quantitation of LC3 immunogold particles at TEM.

### Co-Immunoprecipitation of LC3B-I/II with Proteasome 20S

**Figure 12:**
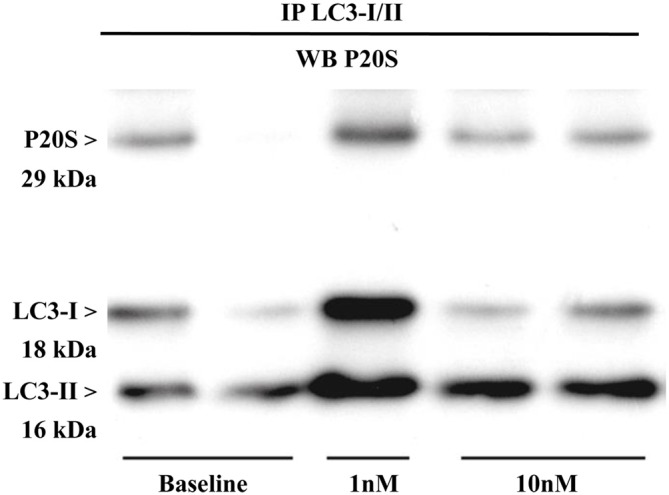
**Immunoprecipitation with anti-LC3 antibody and immunoblotting with anti-P20S**. P20S is clearly detected by western blotting carried out from LC3-immunoprecipitates from two baseline conditions and following two doses of rapamycin (one for 1 nM and two for 10 nM). P20S is present in baseline conditions and following rapamycin. This strongly suggests the occurrence of a molecular binding between LC3 and P20S. The amount of P20S may either increase or decrease depending on how much it is dragged down by LC3, which in turn depends on direct or indirect binding between P20S and LC3 itself which occurs in each experimental conditions. In fact, the intensity of the P20S band does not correspond to P20S particles but it rather represent P20S particles bound to LC3. This phenomenon suggests that the amount of binding between LC3 and P20S decreases when compared with the increase in LC3 and P20S particles *per se*. This is likely to depend on the fact that despite increasing P20S and LC3, mTOR inhibition also promotes the enzymatic conversion of those structures bridging LC3 with P20S as reported in the “Discussion” Section. The representative blotting shows a rapamycin-dependent increase in the ratio between LC3-II and LC3-I which is consistent with rapamycin-induced ATG activation as shown all over the article.

The results presented so far provide a solid evidence for the existence, modulation and quantitation of the novel organelle autophagoproteasome validated at morphological level. However, the presence of two clearing pathways (ATG and UP) within the same organelle does not tell much about their potential interaction. Therefore, in order to assess whether ATG (LC3) and UP (P20S) components interact at molecular level we carried out co-immunoprecipitation experiments. Expression of LC3-II/I was detected in the U87MG cells (Figure 12). Lysates of cells were immunoprecipitated with anti LC3 antibody (3 mg for each sample; Sigma Aldrich, Milan, Italy) and then immunoblotted with anti-P20S (1:100; Abcam).

The P20S was detected in LC3 immunopositive structures of immunoprecipitates from U87MG cells in baseline conditions. These data strongly suggest a molecular binding between LC3 and P20S.

Remarkably, after rapamycin administration LC3-II/I levels dose-dependently increased in immunoprecipitates. In detail, we observed an increase in the ratio between LC3-II and LC3-I which confirms that ATG was activated. In both baseline conditions and following rapamycin, P20S co-immunoprecipitated without any noticeable difference between baseline and rapamycin as shown in representative Figure 12. These latter results may apparently contrast with the well documented increase in the amount of P20S under the effects of rapamycin previously shown. However, it should be kept in mind that an increase in P20S within the cell within the same organelle as LC3 may not correspond to an increase in molecular interactions between LC3 and P20S. Such an interaction may be even reduced under ATG activation. In any case, the significance of these data consist in demonstrating both in baseline conditions as well as following mTOR inhibition that a molecular link binds ATG and UP markers (either within cytosol and/or within vacuoles). The nature of this potential binding is unknown, although it is likely that ubiquitin chains may play a role. Further studies are in progress to assess this intriguing point.

## Discussion

The present study provides a solid morphological evidence indicating that the two major clearing pathways of eukariotic cells (autophagy and proteasome) converge at the level of single organelles named autophagoprotesomes. This is validated by using different gold standard markers for both pathways (LC3 and beclin-1 for ATG; P20S and 19S/PA700, for UP). The co-localization occurs at the level of double or multiple membrane vacuoles. The occurrence of proteasome components within these vacuoles was extensively documented by stoichiometric measurements of each molecule by immunogold-aided TEM. Further evidence was provided at a lower resolution by representative and quantitative confocal microscopy which measured the presence of autophagoproteasomes. The occurrence of autophagoproteasomes is evident already in baseline conditions both at electron and confocal microscopy. However, both techniques indicate a dose-dependent increase in these organelles when the mTOR inhibitor rapamycin is administered. The increase in autophagoproteasomes under the dose-dependent effects of rapamycin is more pronounced at electron compared with confocal microscopy. Such a difference depends on the lower resolution of confocal microscopy compared with TEM, where single proteins merging within a vacuole are detected. In contrast, only high amount of molecules provide a detectable signal at fluorescence confocal microscopy. The occurrence of autophagoproteasomes in baseline conditions is remarkable when considering the experimental setting we selected to carry out the present experiments. In fact, GBM cell lines highly express mTOR, which in turn suppresses ATG (Jiang et al., 2009). We selected on purpose these experimental conditions to hardly challenge our experimental hypothesis. In fact, if the occurrence of autophagoproteasomes as a morphological entity is a noticeable anatomical effects, this should be detectable even when only a few vacuoles are present in the cell. In fact, we demonstrated that autophagoproteasomes can be documented even when mTOR is overactive and ATG is inhibited. This indicates that such an organelle persists even in detrimental ATG conditions. As expected, when ATG was recovered by a dose-dependent mTOR inhibition, the occurrence of autophagoproteasomes blossomed up to higher levels compared with LC3 positive ATG vacuoles observed in baseline conditions. The occurrence of ATG stimulation under the effects of mTOR inhibition induced by rapamycin was confirmed in the present experimental conditions by using a variety of internal controls. In fact, we concomitantly measured an increase of the following items: double-membrane vacuoles, LC3 positive vacuoles, LC3 particles in the cytosol. All these markers measured at TEM are gold standards to identify ATG structures, which were augmented dose-dependently by rapamycin administration. Thus, the autophagoproteasome remarkably overlaps the trend of the cell ATG status and it is equally stimulated by a classic ATG stimulator. Unexpectedly, in this research article we clearly documented that rapamycin, apart from its mTOR-dependent ATG stimulation, strongly and dose-dependently overexpressed UP components. The amount of overexpression of P20S, again, overlapped with LC3 and ATG vacuoles. Altogether these data indicate that cell clearing pathways, which where once considered to be distinct morphological entities undergoing a distinct metabolic regulation, indeed represent a unified morphological clearing apparatus. At this level all components eventually merge sharing at least some common regulators which rely on the status of mTOR activity. The functional merging is not solely grounded on the presence of similar activators or the convergence within multi-membrane structures, but it is strongly implicated by the occurrence of remarkable co-immunoprecipitation of LC3 and P20S. These latter findings rely on either direct or indirect binding between these molecules. So far, no data are available showing all these evidence and no study addressed neither the morphology of autophagoproteasome nor a binding between LC3 and P20S. At present, we cannot provide the molecular basis for such a binding. One might speculate that the key clearance-related protein ubiquitin may be a good candidate to constitute an intermolecular bridge. In fact, both ATG and UP pathways are initiated upon substrate ubiquitination (Clague and Urbé, 2010; Shaid et al., 2013). In keeping with this, it should be considered that, when ATG progresses physiologically the ATG marker LC3 is eventually metabolized, which poses the question whether such a metabolism is carried out upon poly-ubiquitination by UP complex within autophagoproteasomes. Very recently it was demonstrated that, during the native state of ATG organelles, at the level of phagophores UP may engage substrates and terminate protein clearance (Liu and Chen, 2016). A number of articles which were published very recently analyzed the reciprocal interactions between proteasome and autophagy by measuring reciprocal variations of activity within various experimental conditions (Ding et al., 2007; Zhu et al., 2010; Wang et al., 2013; Zhang and Manning, 2015; Zhao et al., 2015). However, none of these articles investigated the occurrence of a merged morphological entity and, in general, no study addressed sub-cellular compartmentalization of such a functional interaction. In this manuscript we specifically defined this compartment as a novel organelle, where hallmarks of both pathways actually merge. From a functional level, despite slight differences it is clear that expression of both ATG and UP markers within these organelles are equally induced by mTOR inhibition. Nonetheless a number of articles provided functional evidence speaking out for the occurrence of proteasome inhibition when ATG is stimulated (Buss et al., 2010; Zhang et al., 2015). This apparently contrasts with present findings where we noticed a concomitant expression of both components. However, the discrepancy might be explained by some of our findings showing that, when ATG is slightly activated there is an imbalance within autophagoproteasome shifting towards an excess of ATG vs. UP components, whereas upon maximal ATG activation the reciprocal phenomenon occurs. In any case, if one considers the absolute amount of ATG and UP proteins, they are both increased following mTOR inhibition, which confirms the recent findings by Zhao et al. (2015) showing that upon mTOR inhibition both ATG and UP are activated. The functional data based on ATG and UP activity are perfectly in line with our morphological findings showing concomitant expression of ATG and UP components following rapamycin. Most importantly, this lends substance to a synergistic empowerment of the autophagoproteasome which is made up by both pathways.

Despite its morphological novelty autophagoproteasome appears to be the natural site where cell clearing pathways (once believed to be anatomically segregated) indeed co-exist and they are likely to interact at molecular level. These data indicate that ATG and UP often represent different facets of a single organelle in which unexpected amount of enzymatic activity is potentially available.

Thus, autophagoproteasome may represent a sophisticated and complex ultimate clearing apparatus which is likely to provide for a variety of physiological activities of the cell and which is recruited by a variety of detrimental triggers.

**Figure 13:**
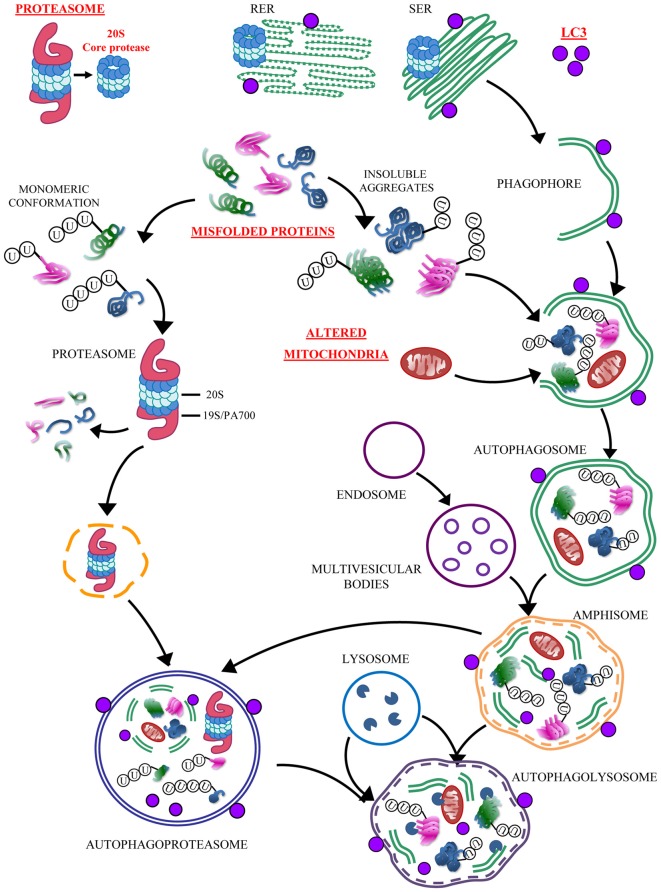
**Convergency between UP and ATG**. The simplified cartoon reports the main clearing pathways of eukaryotic cells emphasizing the significance of the autophagoproteasome which is the focus of the present manuscript. This organelle is likely to play a pivotal role in those disorders which are characterized by accumulation of misfolded proteins and altered mitochondria. The significance of this novel anatomical entity for neurological disorders is likely to be enormous since a growing body of neuropathological and pathobiochemical data demonstrate a failure of clearing pathways as key in both acute and chronic neurodegenerative conditions. These include cerebrovascular disorders and epilepsy (Natale et al., 2011; Orzi et al., 2013; Giorgi et al., 2015). In these disorders a defective ATG or UP are more and more evident (Ge et al., 2012; Caldeira et al., 2014; Fang et al., 2015; Giorgi et al., 2015; Wu et al., 2015). In fact, each step of the UP and/or ATG cascade may be affected either by toxic exposure or genetic mutations. Thus, providing the evidence for a further and ultimate organelle is expected to uncover a variety of disease causes and molecular mechanisms to be deciphered in clinical neuroanatomy. The site-specificity of the occurrence of autophagoproteasomes within different brain areas, and within each area between different neuronal cell type calls for in depth investigations aimed at disclosing the intrinsic vulnerability which may characterize various neural population in the presence of autophagoproteasome failure. In baseline conditions autophagoproteasome does occur. This is true also for extremely detrimental conditions for ATG machinery just like those reported in baseline conditions in the present manuscript. In fact, U87MG cells possess a dramatic upregulation of mTOR which in turn suppresses ATG. It seems that this produces an inhibition of both ATG and UP machinery leading a synergistic suppression of autophagoproteasome. This evidence confirms what recently postulated based on functional studies on the dual regulation of UP and ATG by mTOR (Zhao et al., 2015). Nowadays, owing an improved knowledge of a parallelism between these clearing pathways we are allowed to face the problem as a unified matter where the convergence of both pathways is expected to be dramatically occluded by mTOR inhibition. Nonetheless, even when mTOR is overactive we observed that, in line with the cartoon, the autophagoproteasome “may be derived from the fusion of ubiquitin-proteasome with either early or late autophagosome containing cytosolic material at various stages of degradation” (Klionsky et al., 2016). This adds on enzymatic activity of amphisome thus leading to the most powerful clearing system potentially described in eukaryotic cells so far.

Concerning this point, we wish to mention that the first TEM evidence we obtained serendipitously for the placement of UP components within ATG vacuoles was obtained within an experimental context in which our research team was investigating the electron microscopy effects induced by methamphetamine. This is a powerful drug of abuse and also a neurotoxin which dramatically alters the ATG pathway (Larsen et al., 2002; Fornai et al., 2004; Castino et al., 2008; Pasquali et al., 2008). In fact, methamphetamine produces a massive accumulation of giant aberrant ATG vacuoles (Fornai et al., 2004). In the recent data reported here for the first time the experimental conditions are just opposite since in GBM cell lines ATG is depressed. Nonetheless, the presence of autophagosome was still noticeable. Conversely, in PC12 ATG is upregulated and baseline levels of autophagosomes are elevated (Fornai et al., 2007). This is further augmented by methamphetamine exposure (Fornai et al., 2004, 2007; Lazzeri et al., 2007). Thus, it is not surprising that the density of autophagoproteasome we measured here in PC12 in baseline conditions is two-fold the density we measured in U87MG. In any case, even in U87MG cells where ATG is suppressed, the autophagoproteasome is detectable and it increases dramatically by blocking mTOR with rapamycin. This produces a concomitant increase in autophagosomes and autophagoproteasomes. As reported in the cartoon of Figure 13, “the autophagoproteasome may be derived from the inclusion of ubiquitin-proteasome structures within either early or late autophagosomes containing cytoplasmic material at various stages of degradation” (Klionsky et al., 2016), which adds on enzymatic activity of amphisome which leads to the most powerful clearing system described in eukaryotic cells so far. Assuming that the thickness of the ultrathin slide allows to visualize a small part of the whole volume of each vacuole which is stained by small particles, we assume that the amount of autophagoproteasomes in the cell reported here is way underestimated. For the same reason, it is likely that all vacuoles described here contains LC3 since these particles were evident in roughly 50% of the sections. At the same time, the occurrence of P20S, which is likely to be more scattered was detected in 12% of the vacuole areas. If we assume that each ATG vacuole is indeed an autophagoproteasome than we would expect that the amount of vacuoles containing both LC3 and P20S in the same ultrathin section corresponds to the combination of the chances to visualize either LC3 or P20S. In fact, this chance roughly corresponds to the number of double stained vacuoles we counted. Remarkably, the ratio between the number of clearance hallmarks (LC3 and P20S) and the number of vacuoles remains steady for the whole range of ATG activation. This suggests that, when ATG is occluded the ambiguous nature of the vacuoles (both ATG and UP organelles) is not substantially modified. This will be consistent with the hypothesis that ATG stimulation necessarily implies proteasome activation and vice versa, in contrast ATG inhibition (or UP inhibition) is expected to occlude both phenomena, at the same time for the same magnitude. Recent functional evidence on this point provides contrasting data (Korolchuk et al., 2009; Wang et al., 2013; Tian et al., 2014; Zhang et al., 2015; Zhao et al., 2015). This may depend on the slight shifting we documented here between mild and strong mTOR inhibition which produces a slight prevalence of ATG over UP or vice versa, respectively. In line with this hypothesis one may speculate that the pivotal molecular complex for ATG regulation, mTOR, is pivotal for UP regulation as well, but fine modulation is critical. This experimental issue is presently under scrutiny in our laboratory.

## Conclusion

In the present manuscript evidence is provided that ATG and UP components are placed in a novel organelle, which is now detailed at morphological level. This is the site where a functional interaction between ATG components and UP takes place to terminate substrate degradation as very recently postulated by Liu and Chen (2016).

## Author Contributions

PL carried out electron microscopy and wrote the article. GL carried out cell cultures. FB carried out immonoprecipitation. CLB critically revised the work. SG critically revised the work for intellectual content. AS carried out confocal microscopy. FF designed the work and wrote the article.

## Funding

Ricerca Corrente 2016 Ministero della Salute.

## Conflict of Interest Statement

The authors declare that the research was conducted in the absence of any commercial or financial relationships that could be construed as a potential conflict of interest. The reviewer MF declared a shared affiliation, though no other collaboration, with several of the authors PL, GL, AS, FF to the handling Editor, who ensured that the process nevertheless met the standards of a fair and objective review.
